# The Role of Micronutrients in Atherosclerosis: Mechanisms and Clinical Application

**DOI:** 10.1111/jcmm.70920

**Published:** 2025-10-29

**Authors:** Yuxin Ouyang, Weiwei Jiang, Xiongquan Long, Peng Mao, Pingping He, Xinping Ouyang

**Affiliations:** ^1^ Department of Physiology, School of Basic Medicine Hunan Normal University Changsha Hunan China; ^2^ Department of Physiology, Institute of Neuroscience Research, Hengyang Key Laboratory of Neurodegeneration and Cognitive Impairment, College of Basic Medicine Hengyang Medical College, University of South China Hengyang Hunan China; ^3^ Department of Organ Transplantation Zhujiang Hospital, Southern Medical University Guangzhou Guangdong China; ^4^ Department of Gastroenterology The First Affiliated Hospital of Hunan Normal University (Hunan Provincial People's Hospital) Changsha Hunan China; ^5^ Aging Health Research Center, School of Nursing Hunan Normal University Health Science Center Changsha Hunan China; ^6^ Key Laboratory of Model Animals and Stem Cell Biology in Hunan Province, Hunan Normal University School of Medicine; Engineering Research Center of Reproduction and Translational Medicine of Hunan Province Manufacture‐Based Learning and Research Demonstration Center for Human Reproductive Health New Technology of Hunan Normal University Changsha Hunan China

**Keywords:** atherosclerosis, inflammation, low‐density lipoprotein, micronutrients, minerals, vitamins

## Abstract

Micronutrients, though required in relatively small quantities by the human body, are essential for maintaining normal physiological functions and play a crucial role in the prevention and management of various diseases. Atherosclerosis (AS) is a common chronic inflammatory condition that often presents without obvious symptoms in its early stages but can lead to severe health issues such as acute myocardial infarction and stroke. The involvement of micronutrients in the early prevention and treatment of AS is critical, yet the efficacy of micronutrient supplementation for AS remains a subject of debate, and the specific mechanisms by which micronutrients influence AS are not fully understood. This study systematically summarises the mechanisms of micronutrients in AS and proposes that their roles in AS prevention and treatment should be properly understood and utilised. We further point out the limitations of current research and propose the future direction of systemic interventions based on the nutritional network, providing novel strategies for the prevention and treatment of AS.

## 1 Introduction

Atherosclerosis (AS) represents a complex disease characterised by chronic inflammation and disruptions in lipid homeostasis, primarily manifesting in medium and large arteries. It serves as a common pathological precursor to cardiovascular diseases (CVD) [1]. Initiated by endothelial damage, AS triggers the migration of lipid proteins (Low‐Density Lipoprotein, cholesterol, etc.) into blood vessels, leading to their adsorption onto vessel walls. In the intima, low‐density lipoprotein cholesterol (LDL‐C) undergoes oxidation to form oxidised low‐density lipoprotein (ox‐LDL). Ox‐LDL has a strong proinflammatory effect and stimulates the release of proinflammatory cytokines as well as adhesion factors from endothelial and smooth muscle cells in the arterial wall [2]. At the same time, blood monocytes migrate and adhere to the intima and differentiate into macrophages, which phagocytose excess ox‐LDL to form lipid‐filled foam cells that gradually accumulate in the vessel wall [3]. Foam cells not only mark the early lesions of AS, but also further exacerbate the inflammatory response by releasing various pro‐inflammatory substances such as tumour necrosis factor‐α (TNF‐α), interleukin‐1 (IL‐1) and interleukin‐6 (IL‐6) [4]. The combination of the inflammatory response and lipid accumulation leads to plaque formation [5]. Subsequent progression of AS may eventually lead to luminal stenosis, plaque calcification, bleeding or rupture, triggering an acute cardiovascular event or even death [6]. Although AS is chronic, it is usually asymptomatic until acute and life‐threatening symptoms develop. Therefore, mitigating the risk factors associated with AS and impeding its progression are important areas of current research.

The current understanding of the pathogenesis of AS encompasses multiple factors, including dyslipidemia, chronic inflammation, endothelial dysfunction and gene–environment interactions, which have led to the development of integrated therapeutic strategies combining various approaches [7]. Current clinical management primarily relies on pharmacological treatments (e.g., statins, β‐blockers, angiotensin receptor blockers), interventional procedures, surgical interventions and lifestyle modifications [8]. While these approaches collectively reduce the incidence of major adverse cardiovascular events, significant limitations remain. Pharmacotherapy, though effective, often carries side effects and long‐term adherence challenges. Post‐interventional therapy typically requires prolonged antiplatelet medication and carries risks of vascular restenosis [9]. Surgical options involve substantial risks, extended recovery periods and the possibility of AS progression even after intervention. Notably, among all modifiable factors, dietary intervention has attracted significant attention due to its sustainability and multi‐target effects. In this context, the critical role of micronutrients in the pathophysiology of AS has been increasingly recognised [10]. Although the human body requires these substances in minimal amounts, they serve as key mediators in antioxidant, anti‐inflammatory and metabolic homeostasis processes, regulating the progression of AS through multiple molecular pathways [11]. Current research suggests that the pleiotropic mechanisms of micronutrients may provide novel insights into understanding AS, though further high‐quality evidence is still needed to clarify their clinical translational value.

Nutrients can be broadly classified into macronutrients and micronutrients based on their required quantities within the body [12]. Macronutrients—carbohydrates, lipids and proteins. Micronutrients, often termed essential nutrients, are indispensable as they cannot be synthesised endogenously and must be sourced from food [13]. Micronutrients play indispensable roles in diverse physiological processes, including growth, energy metabolism, immune regulation and coagulation function [14]. However, current research predominantly focuses on individual micronutrients, failing to adequately elucidate their complex interaction networks (e.g., synergistic or antagonistic effects). This knowledge gap significantly limits their precise application in AS. By synthesising emerging evidence, we systematically characterise how micronutrients modulate key AS pathological mechanisms (including oxidative stress, inflammatory regulation and endothelial protection), emphasising their interrelationships. Building upon these insights, we propose a systematic nutritional intervention strategy designed to overcome the limitations of single‐nutrient research, which not only provides a theoretical foundation for personalised nutritional therapy and dietary prevention of AS but also establishes new directions for future precision nutrition research focusing on multi‐nutrient synergies.

## 2 Methods: Literature Search and Selection

The literature for this review was identified through a combination of systematic database searches and expert selection. We searched PubMed, Web of Science and Scopus up to September 2025 using the following core keyword combination: (‘micronutrient*’ OR ‘vitamin*’ OR ‘mineral*’ OR ‘trace element*’) AND (‘atherosclerosis’ OR ‘arteriosclerosis’ OR ‘plaque’ OR ‘cardiovascular disease’). The final selection of studies was based on their relevance to the topic, scientific rigour and academic impact. It was further supplemented by reference tracing to ensure comprehensive coverage of key research.

## 3 Micronutrients and AS

The World Health Organization defines micronutrients as nutrients measured only in micrograms or milligrammes that are required for normal physiological functioning of the human body [15]. Micronutrients mainly include vitamins and minerals. Vitamins can be further categorised into fat‐soluble vitamins (including vitamins A, D, E and K) and water‐soluble vitamins (including B vitamins and vitamin C) based on solubility characteristics, and minerals can be categorised into macrominerals (including calcium, phosphorus, potassium, sodium, magnesium and sulphur) and trace minerals (including iron, zinc, copper, manganese, iodine and selenium) [16]. There exists an intricate and delicate dynamic equilibrium between micronutrients and AS. Although these micronutrients are present in minute quantities in the human body, they collectively maintain cardiovascular homeostasis through synergistic interactions. Deficiency or excess of any single micronutrient can disrupt physiological equilibrium, subsequently promoting AS development by affecting multiple pathophysiological mechanisms, including oxidative stress, inflammatory responses and endothelial dysfunction [17].

In physiological terms, micronutrients are integral to normal metabolic processes and contribute to vascular health through multiple pathways. Importantly, their effects vary across different stages of atherosclerotic development. In the initiation phase, vitamins C and E act as potent antioxidants that neutralise free radicals, thereby preventing oxidative modification of LDL and reducing endothelial injury [18, 19, 20]. Zinc and selenium, as essential cofactors of superoxide dismutase and glutathione peroxidase, strengthen endogenous antioxidant defences and inhibit foam cell formation at its earliest stage. During disease progression, vitamin D regulates macrophage and T‐lymphocyte activity, suppressing the release of pro‐inflammatory cytokines such as TNF‐α and IL‐6, thereby mitigating chronic plaque inflammation [21]. Vitamin B3 (niacin) contributes to lipid metabolism regulation by lowering triglyceride and lipoprotein(a) levels [22]. Vitamin K, through activation of matrix Gla protein (MGP), inhibits aberrant vascular calcification while promoting extracellular matrix synthesis, thus supporting fibrous cap stability [23]. In the complication stage, vitamin E exerts antithrombotic effects by reducing platelet aggregation and adhesion [24]. Magnesium, acting as a natural calcium channel blocker, regulates vascular tone while limiting excessive vascular smooth muscle cell proliferation and calcification [25]. Additionally, vitamin K further prevents pathological calcium deposition in vessel walls by modulating calcium metabolism, thereby enhancing plaque stability and lowering the risk of rupture [26]. These micronutrients intervene in the progression of AS at different pathological stages through multi‐targeted, stage‐specific synergistic mechanisms. Recent studies have found that micronutrients can indirectly influence the course of AS by modulating gut microbial composition and function [27]. Gut microbes metabolise micronutrients (e.g., vitamin K, B vitamins), which can generate biologically active metabolites (e.g., short‐chain fatty acids, SCFAs), and SCFAs further exert anti‐AS effects by inhibiting inflammation, improving endothelial function and regulating lipid metabolism [28, 29]. From a pathological point of view, micronutrient homeostasis is closely linked to the development of AS. When iron is in excess, a large number of hydroxyl radicals are produced, exacerbating oxidative stress and promoting LDL oxidation and vascular endothelial damage; whereas iron deficiency may lead to insufficient haemoglobin synthesis, triggering tissue hypoxia and stimulating vascular smooth muscle cell proliferation [30]. The imbalance in the ratio of micronutrients such as zinc and copper will affect the activity of metalloproteinases, destabilise the vascular wall and promote plaque rupture [31]. In addition, in the process of homocysteine metabolism, the lack of vitamin B12, B6 and folic acid will lead to the accumulation of homocysteine in the body, damage to the endothelial cells of the blood vessels, promote platelet adhesion and aggregation and accelerate the formation of blood clots [32].

In clinical studies, there is inconsistency in findings regarding the relationship between micronutrients and AS. Systematic evaluations of observational studies indicate that individuals in low‐selenium regions exhibit significantly higher risks of CVD. Although some randomised controlled trials suggest selenium supplementation may improve lipid profiles and endothelial function, these benefits are strictly dependent on baseline selenium status, and high‐quality evidence remains insufficient [33]; however, for individuals at high risk of CVD, large randomised controlled trials have demonstrated that daily supplementation with 400 IU of natural vitamin E over an average follow‐up period of 4.5 years did not reduce the risk of the primary composite endpoint of myocardial infarction, stroke or cardiovascular death (relative risk 1.05). This conclusion is highly reliable due to the trial's rigorous design and large sample size [34]. On the one hand, such differences may be due to individual genetic polymorphisms, such as different genotypes of the vitamin D receptor gene (VDR), which may affect the efficiency of vitamin D absorption and utilisation, leading to individual differences in supplementation [35]; on the other hand, a variety of confounding factors, such as dietary structure, lifestyle and so on, may interfere with the final results of the study. Furthermore, interactions between micronutrients add another layer of complexity to their relationship with AS. For instance, vitamin C enhances iron absorption and potentiates the antioxidant effects of vitamin E [36]. Conversely, copper and zinc exhibit antagonistic interactions in enzyme regulation—excessive zinc intake inhibits copper absorption, disrupting their balance and impairing the function of copper‐dependent enzymes, which play critical antioxidant and anti‐atherosclerotic roles [37]. However, current research on micronutrient interactions remains limited. Studies focusing on single micronutrients often overlook their potential effects on other nutrients, leading to oversimplified conclusions that require further investigation and clinical validation. A deeper understanding of each micronutrient's mechanism of action, combined with systematic analysis of their interactions, is essential for developing effective nutritional intervention strategies.

## 4 The Mechanism of Micronutrients With Notable Effects on AS

**FIGURE 1:**
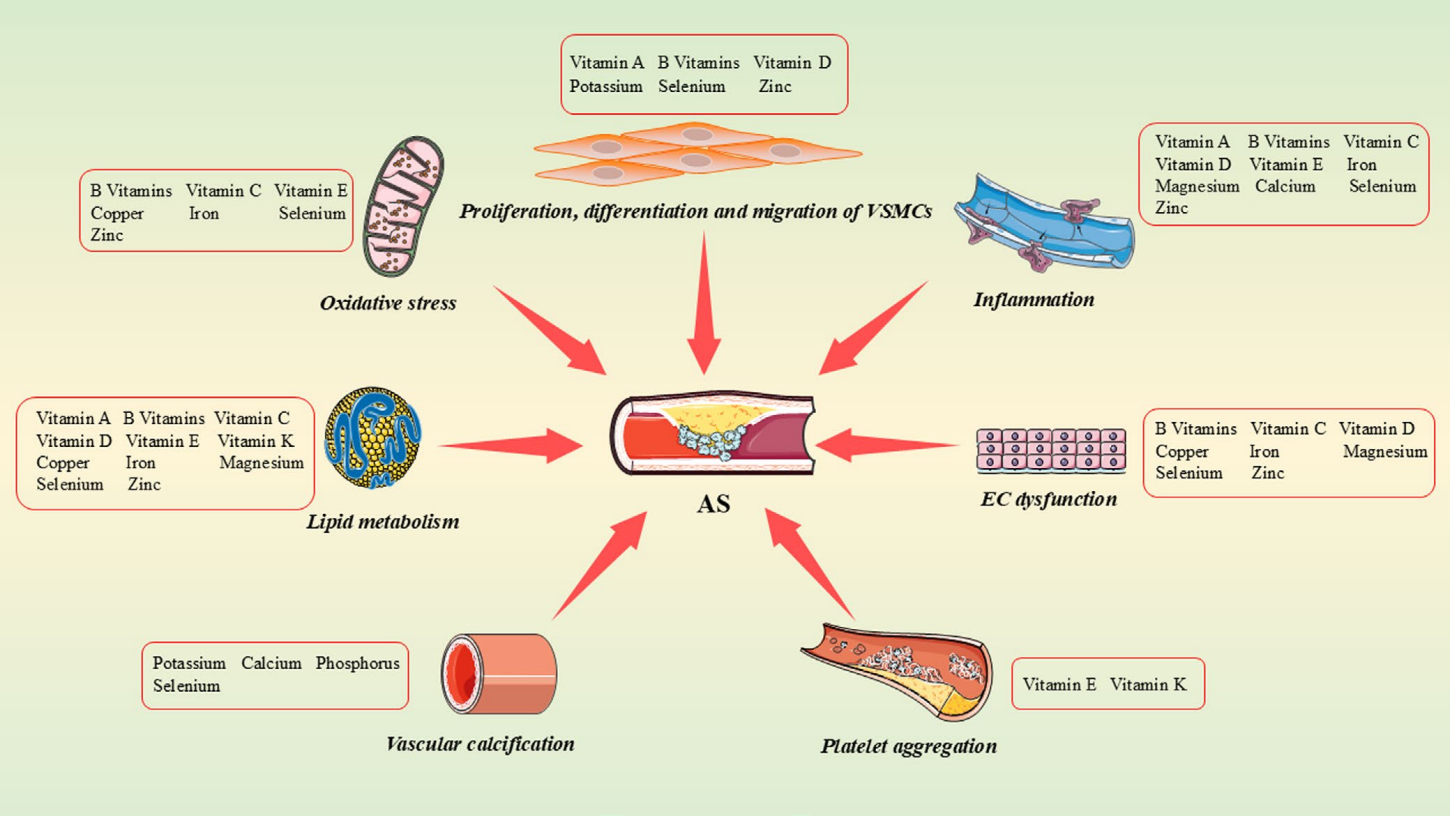
Micronutrients are involved in the key biological pathway of atherosclerosis (AS). Micronutrients play a crucial role in numerous biological pathways that contribute to the pathophysiological processes of AS. These pathways include lipid metabolism, vascular calcification, oxidative stress, proliferation, differentiation and migration of vascular smooth muscle cells (VSMCs), inflammation, endothelial cell (EC) dysfunction and platelet aggregation.

Micronutrients play a pivotal role in the development and progression of AS, exerting protective effects through a complex network of molecular mechanisms (Figure 1). Recent studies indicate that, despite differences in chemical structures and metabolic pathways, various micronutrients can form synergistic regulatory networks by targeting common signalling pathways. In antioxidant defence, selenium, zinc and vitamins C and E collectively activate the Nrf2 signalling pathway. Through distinct mechanisms, they synergistically enhance the activity of endogenous antioxidant enzymes, effectively mitigating vascular oxidative damage [38, 39, 40]. Regarding inflammation regulation, zinc, vitamins D and B6 inhibit nuclear factor kappa B (NF‐κB) signalling. Zinc maintains the activity of the deubiquitinating enzyme A20 [41], vitamin D binds to the vitamin D receptor to interfere with NF‐κB transcription [42], and vitamin B6 inhibits IKK complex phosphorylation [43]. Together, these micronutrients suppress NF‐κB activation at multiple points, effectively reducing the expression of pro‐inflammatory cytokines such as TNF‐α and IL‐6. In maintaining endothelial function, magnesium, vitamins and selenium can act on the Akt/eNOS signalling axis, enhancing nitric oxide (NO) bioavailability and improving endothelium‐dependent vasodilation through complementary mechanisms [44]. Magnesium directly activates eNOS as a cofactor [45], vitamin C prevents eNOS uncoupling by preserving tetrahydrobiopterin in its reduced state [46], and selenium protects eNOS function through its antioxidant activity [47]. Collectively, these actions enhance NO bioavailability and promote vascular homeostasis. This integrated, synergistic mechanism not only deepens our understanding of the cardiovascular protective effects of micronutrients but also provides a theoretical foundation for precision nutritional interventions targeting AS. It underscores the importance of emphasising combined micronutrient supplementation in clinical practice to achieve optimal prevention and treatment outcomes.

In the following, we will summarise and subdivide the specific mechanisms of action of different vitamins (including A, B‐complex, C, D, E, K, etc.) and minerals (e.g., iron, zinc, selenium, etc.) in AS, providing the fundamental theoretical basis for precise nutritional interventions. Still, it is worth noting that this does not imply that these nutrients can act independently. There is a complex synergistic or antagonistic relationship between them. It is because of this interdependence that the effects of supplementing with a particular nutrient in isolation are often constrained by the overall nutritional status [48]. In the following discussion, we will not only analyse the specific mechanisms of individual micronutrients but also focus on their interactions, thereby establishing a theoretical foundation for developing more effective, comprehensive nutritional strategies.

### 4.1 Vitamins and AS

#### 4.1.1 Vitamin A

Vitamin A primarily exists in the diet as preformed forms (e.g., retinol, retinyl esters) and as precursors (e.g., β‐carotene), and its role in AS remains complex and somewhat controversial [49]. Evidence suggests that vitamin A may influence AS through multiple mechanisms, including antioxidant activity, modulation of immune responses, regulation of gene expression, inhibition of vascular smooth muscle cell (VSMC) proliferation and improvement of lipid metabolism. At the molecular and cellular levels, vitamin A and its derivatives (e.g., all‐trans retinoic acid) can alleviate endothelial dysfunction, regulate T‐cell polarisation and cytokine secretion, influence macrophage phenotype switching and modulate VSMC proliferation, differentiation and migration [50, 51]. Recent animal studies indicate that β‐carotene promotes regression of atherosclerotic plaques in mice, potentially by increasing the number of regulatory T cells within plaques [52]. Additionally, Buosa et al. [53] found in a cross‐sectional study (DIABIMCAP cohort) that plasma concentrations of β‐carotene and total carotenoids were negatively correlated with VLDL and its subtypes, and that plasma total carotenoid concentrations were significantly lower in patients with AS compared to non‐AS patients. This study employed an observational design with a limited sample size (*n* = 204) and did not involve randomised grouping. However, existing high‐quality evidence does not demonstrate clear benefits regarding vitamin A's role in preventing CVD. A meta‐analysis of multiple large randomised controlled trials indicates that supplementation with vitamin A alone or other antioxidant vitamins does not significantly prevent major cardiovascular events, with consistent conclusions even in studies of higher methodological quality. Some studies even suggest that high‐dose vitamin A may increase cardiovascular risk. These findings are corroborated by a larger 2018 systematic review of nearly 180 RCTs, which revealed that common dietary supplements (including multivitamins, vitamin D, calcium and vitamin C) had no significant impact on cardiovascular events or all‐cause mortality. Certain supplements, such as niacin (combined with statins) and vitamin A‐containing antioxidant complexes, may slightly increase all‐cause mortality risk [54, 55]. These existing evidences indicate that supplements like vitamin A do not provide additional cardiovascular protection in nutritionally adequate populations. It is recommended that nutritional needs be met primarily through a balanced diet. Some interventional studies suggest potential benefits: for example, β‐carotene significantly inhibits AS progression in ApoE‐deficient mice and corrects vitamin A deficiency, while retinoic acid effectively suppresses vascular cell calcification in humans [56, 57]. Moreover, combined supplementation with vitamins A and D reduces serum IL‐1β levels and improves clinical outcomes in ischemic stroke patients [58]. Overall, current evidence suggests that vitamin A may contribute to the prevention and treatment of AS, although its efficacy likely depends on chemical form, co‐administered nutrients and individual nutritional status. Future large‐scale, long‐term clinical trials are warranted, particularly to investigate differences among vitamin A forms and their potential benefits in specific populations.

#### 4.1.2 B Vitamins

**FIGURE 2:**
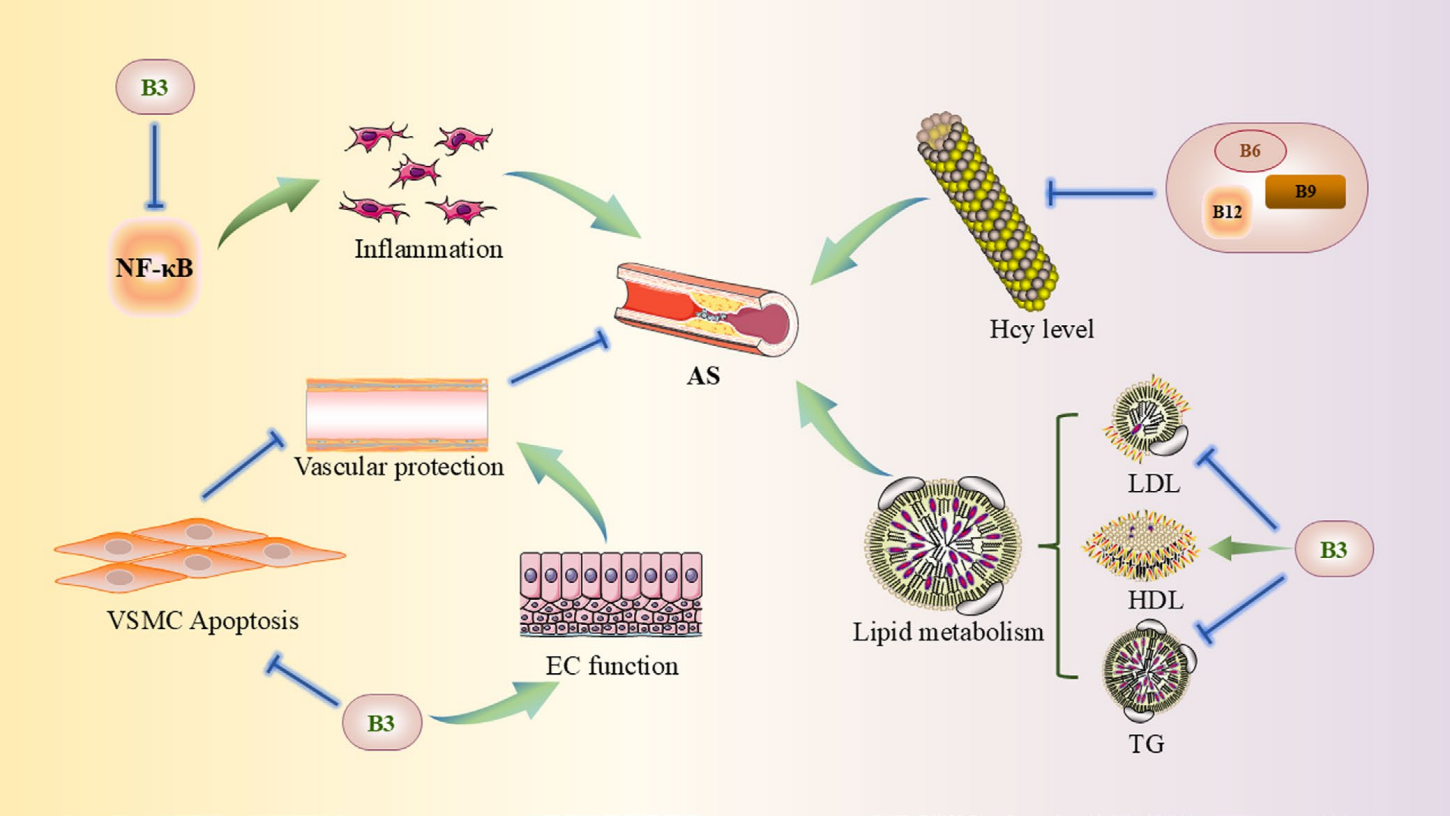
Proposed interplay between b vitamins and key pathways in atherosclerosis. This diagram outlines the role of B vitamins in atherosclerosis (AS). Vitamin B3 exerts its effects through multiple mechanisms: Inhibiting the pro‐inflammatory NF‐κB pathway to reduce inflammation; suppressing vascular smooth muscle cell (VSMC) apoptosis; enhancing endothelial cell function; and promoting vascular protection. Additionally, B3 aids in regulating lipid metabolism by lowering lipoprotein (LDL) and triglycerides while increasing high‐density lipoprotein (HDL). Deficiencies in vitamins B6, B9 and B12 elevate homocysteine(Hcy), a key AS risk factor that disrupts lipid balance and vascular integrity. These interactions underscore the complex significance of B vitamins in AS.

B vitamins comprise various water‐soluble vitamins, including B1, B2, B3, B6, B12 and folic acid (Figure 2). These vitamins are vital for cellular metabolism, energy production, red blood cell formation and nervous system function [59]. Elevated levels of Hcy, a sulphur‐containing amino acid, are recognised as risk factors for CVD and cognitive impairments [60, 61]. Elevated Hcy contributes to AS through mechanisms such as lipid metabolism disorders [62], accumulation of reactive oxygen species (ROS) and endoplasmic reticulum (ER) stress [63, 64], homocysteinylation of haemoglobin and ferritin [65], endothelial dysfunction [66, 67], increased vascular wall inflammation [68], enhanced interaction with lipoprotein(a) (Lp(a)) [69], reduced hydrogen sulphide production [70] and increased platelet adhesion and aggregation [71]. Vitamins B6, B9 and B12 are crucial for Hcy metabolism: B9 and B12 are involved in remethylating Hcy to methionine, while B6 facilitates its conversion to cysteine via the transsulfuration pathway [72]. Andrews et al. [73] demonstrated in apoE‐deficient mice that a diet low in methyl donors and B vitamins increased aortic plaque burden compared to a control diet. Additionally, a prospective study indicated that foetuses exposed to lower circulating levels of vitamin B12 had increased carotid intima‐media thickness (cIMT) in childhood, an early indicator of AS [74]. Since plant‐based foods lack vitamin B12, animal products are the primary dietary sources of this vitamin [75]. A double‐blind crossover study revealed that vitamin B12 supplementation significantly improved brachial artery flow‐mediated dilation (FMD) and cIMT in vegetarians compared to a placebo group [76]. For subjects with baseline Hcy levels of 9.1 μmol/L or higher, those randomised to receive a combination of vitamins B (5 mg folic acid + 0.4 mg vitamin B12 + 50 mg vitamin B6) showed a significant reduction in the rate of cIMT progression compared to the placebo group. However, for subjects with baseline Hcy levels below 9.1 μmol/L, vitamin B supplementation was not significantly effective, suggesting that B vitamin supplementation may reduce subclinical AS progression by lowering Hcy levels [77]. Despite the link between elevated Hcy and CVD, the efficacy of regular B vitamin supplementation (B6, B9 and B12) in reducing CVD risk remains contentious. Many large randomised controlled trials have not supported this hypothesis, showing no significant effect of B vitamin supplementation on CVD, myocardial infarction (MI), coronary heart disease or all‐cause mortality. The Heart Outcomes Prevention Evaluation‐2 (HOPE‐2) trial, the Western Norway B Vitamin Intervention Trial (WENBIT) and the Norwegian Vitamin Trial (NORVIT) all reached similar conclusions [78, 79, 80]. However, a meta‐analysis indicated that in regions without or with partial folic acid fortification, combined supplementation of folic acid, vitamin B12 and vitamin B6 significantly reduced stroke risk, whereas no benefit was observed in fortified regions [81]. These findings suggest that the potential benefits of B vitamins may be limited to specific populations (e.g., elderly individuals, vegetarians, pregnant/lactating women and other groups prone to nutritional deficiencies), while showing minimal effects in those with adequate nutritional status. Furthermore, the metabolism of B vitamins can be influenced by other micronutrients (e.g., zinc)—zinc deficiency may impair the conversion to their active forms [61], highlighting the need to consider synergistic interactions among nutrients. In summary, current evidence does not support using B vitamins as a universal therapeutic intervention for AS. However, their potential role in adjunctive management for high‐risk subpopulations warrants individualised evaluation.

Niacin, also known as vitamin B3 or nicotinic acid, is a water‐soluble vitamin involved in regulating lipid metabolism, inflammation and oxidative stress, thus influencing AS development and progression. Initially described as a lipid‐lowering agent in 1955, niacin can reduce total cholesterol (TC), triglycerides (TG), VLDL, LDL and Lp(a) levels, while increasing high‐density lipoproteins (HDL) and ApoA1 levels at pharmacological doses [82, 83]. In studies with LDLr^−/−^ mice, niacin has been shown to promote macrophage lysosomal cholesterol efflux via the CD38/NAADP signalling pathway, thereby exerting anti‐atherosclerotic effects [84]. Additionally, niacin independently inhibits vascular inflammation, enhances plaque stability and prevents endothelial dysfunction, further contributing to its anti‐atherosclerotic effects [85]. Ganji et al. [86] found that niacin significantly inhibits the production of ROS, the oxidation of LDL and the expression of vascular cell adhesion molecule‐1 (VCAM‐1) and monocyte chemoattractant protein‐1 mRNA, as well as the adhesion of monocytes to ECs in human aortic ECs, which are key events in the development of AS. In ApoE^−/−^ mice, niacin exerts anti‐atherosclerotic effects by inhibiting vascular inflammation through the NF‐κB signalling pathway and reducing VSMCs apoptosis via the Focal Adhesion Kinase signalling pathway [87]. A cross‐sectional study by Agraib et al. demonstrated that increased dietary intake of vitamins B1, B2, B3 and B6 was significantly associated with improved obesity indices and cardiac function parameters in healthy individuals, suggesting that routine B‐vitamin supplementation may mitigate obesity‐related coronary heart disease risk through metabolic modulation [88]. In addition, clinical studies have shown that in patients with coronary heart disease or equivalent risk, the combination of niacin and statin therapy significantly increases HDL levels, decreases LDL and TG levels and reduces both mean and maximum cIMT, outperforming the combination of statins and ezetimibe [89, 90]. However, the clinical use of extended‐release niacin in combination with statins for treating AS remains debatable. Increasing evidence suggests that niacin does not significantly reduce cardiovascular events despite raising plasma HDL‐C (high‐density lipoprotein cholesterol) and lowering LDL‐C and triglyceride levels. In a randomised controlled trial involving patients with dyslipidemia and coronary heart disease, Boden et al. [91] found no significant difference in the incidence of major composite endpoints (such as MI, ischemic stroke and acute coronary syndrome) between the niacin‐statin combination group and the statin‐placebo group, with the niacin combination group experiencing a higher incidence of ischemic stroke. Similarly, a meta‐analysis indicated that niacin combined with statins increased all‐cause mortality risk [92]. Compared to statin monotherapy, the niacin‐statin combination did not lower levels of HDL‐associated apolipoproteins APOC1, APOC2, APOC3 and APOC4. Instead, niacin significantly increased HDL‐associated phospholipid transfer protein, clusterin and haptoglobin/haptoglobin‐related protein levels, which are closely associated with increased AS risk, potentially explaining why niacin treatment does not reduce cardiovascular events [93]. The synthesis of novel niacin derivatives may be an effective approach to overcoming adverse drug reactions in the future. Recent studies have found that simvastatin/niacin‐loaded magnetic nanoparticles, as a drug delivery system, exhibit no significant toxicity in cell experiments and effectively modulate macrophage polarisation [94]. Additionally, the newly developed niacin‐lipoic acid dimer shows promising therapeutic effects in regulating lipid levels and inhibiting AS, effectively reducing niacin‐induced vasodilation [95].

#### 4.1.3 Vitamin C

Vitamin C (ascorbic acid), a key water‐soluble antioxidant, exerts protective effects against the progression of AS through multiple pathways. Its mechanisms extend beyond antioxidant activity to include epigenetic regulation, modulation of cellular signalling pathways and maintenance of vascular structure. Regarding antioxidant and lipid regulation, vitamin C scavenges free radicals and reduces oxidative stress intermediates, effectively inhibiting LDL oxidation and limiting the formation of ox‐LDL. This, in turn, suppresses lipid uptake by macrophages, thereby inhibiting the formation of foam cells [96]. Additionally, studies suggest that vitamin C may downregulate apolipoprotein A (ApoA) expression via TET2‐dependent DNA demethylation, thereby modulating lipoprotein metabolism at the epigenetic level [97, 98]. Although meta‐analyses of large randomised controlled trials (RCTs) in the general population demonstrate limited effects of vitamin C supplementation on the overall lipid profile (with negative primary outcomes), suggesting unclear preventive benefits for broad populations, subgroup analyses and meta‐regression from these studies point to potential significant improvements in lipid parameters in specific subgroups with baseline dyslipidemia or vitamin C deficiency. This conclusion is based on a systematic assessment of multiple RCTs that employed the modified Jadad scale for bias evaluation; however, limitations persist due to the sample sizes and heterogeneity of the included studies [99]. In terms of vascular endothelial protection and functional maintenance, vitamin C has been shown to improve endothelial function. It promotes endothelial cell proliferation and repair by regulating ERK1/2 and ERK5 signalling pathways, thereby enhancing endothelium‐dependent vasodilation [100, 101]. Moreover, vitamin C strengthens basement membrane integrity by promoting the synthesis and deposition of type IV collagen, reducing vascular wall permeability and delaying AS progression [102, 103]. Notably, vitamin C also exhibits synergistic effects with other micronutrients. For example, in intervention studies combining vitamin C with vitamin B12 supplementation, vitamin C enhanced the biological effects of B12, significantly improving cardiovascular risk indicators such as diastolic blood pressure and serum creatinine levels [104]. These findings suggest that future research should further explore the role of vitamin C within composite nutrient strategies, with particular attention to its targeted application in specific risk populations, including the elderly and individuals with hypercholesterolemia. This would provide a scientific basis for the development of precision nutritional intervention programmes.

#### 4.1.4 Vitamin D

Vitamin D, as a steroid hormone, regulates multiple biological processes associated with AS through its active form, 1,25‐dihydroxyvitamin D (1,25(OH)_2_D). By binding to the nuclear vitamin D receptor (VDR), it modulates inflammation, immune responses, endothelial function, lipid metabolism and other pathways [105]. At the molecular and cellular levels, vitamin D exhibits multi‐pathway anti‐atherosclerotic potential. It inhibits foam cell formation, enhances macrophage cholesterol efflux and promotes HDL‐mediated cholesterol transport [106, 107]. Simultaneously, vitamin D delays VSMC senescence by regulating their proliferation, migration and calcification and by inhibiting angiotensin II signalling [108, 109, 110]. Recent studies further reveal that vitamin D reduces plaque volume by mitigating oxidative LDL‐induced endothelial dysfunction and ferroptosis through regulation of medullin expression and activation of the AMPK signalling pathway [111]. Clinical observational studies consistently demonstrate significantly reduced circulating 1,25(OH)_2_D levels in patients with severe coronary AS. Low vitamin D status is closely associated with increased cIMT and impaired flow‐mediated dilation (FMD) [112, 113, 114]. Regarding the effects of vitamin D supplementation on CVD, a large meta‐analysis incorporating 21 RCTs involving over 83,000 participants showed that vitamin D supplementation was not associated with reduced risks of major adverse cardiovascular events, myocardial infarction, stroke, CVD mortality or all‐cause mortality [115]. This unequivocally negative conclusion from the large RCT meta‐analysis is supported by studies in specific disease populations. For example, a randomised double‐blind trial involving 365 elderly men with severe aortic valve calcification further confirmed that daily supplementation with high‐dose vitamin K_2_ (MK‐7) and vitamin D for 24 months did not slow the progression of aortic valve calcification [116]. Furthermore, a Mendelian randomisation study involving over 450,000 individuals provided causal inference evidence from a genetic perspective, indicating no causal association between vitamin D levels and multiple atherosclerotic phenotypes, including coronary heart disease and blood pressure [117]. These three studies collectively form an evidence chain refuting the preventive role of vitamin D in atherosclerotic cardiovascular disease. Taken together, the evidence indicates that the relationship between vitamin D and AS may be influenced by multiple factors, including confounding variables, baseline nutritional status, individual VDR polymorphisms and disease stage. Future research should aim to identify potentially responsive subgroups and further explore non‐classical mechanisms of vitamin D in vascular protection, thereby providing robust evidence to support personalised nutritional interventions.

#### 4.1.5 Vitamin E

Vitamin E, a class of fat‐soluble antioxidants, primarily comprises structural analogues such as tocopherols and tocotrienols, with α‐tocopherol being the most widely distributed in nature and exhibiting the highest biological activity [118]. It exerts multiple protective mechanisms in the prevention and treatment of AS. At the molecular level, vitamin E inhibits lipid peroxidation, reduces the formation of ox‐LDL and downregulates the expression of the scavenger receptor CD36, thereby suppressing macrophage lipid uptake and foam cell formation [119, 120, 121]. Notably, unlike water‐soluble vitamin C, which is mainly localised in the cytosol, vitamin E is concentrated in cell membranes and lipoprotein particles, specifically targeting lipid peroxidation chain reactions. This property endows it with a unique role in preserving the integrity of biological membranes. Additionally, vitamin E exhibits anti‐inflammatory effects by inhibiting protein kinase C (PKC) activity in monocytes and reducing the secretion of pro‐inflammatory cytokines, including IL‐1β and TNF‐α [122, 123, 124, 125]. Furthermore, it suppresses platelet aggregation through PKC‐dependent pathways, demonstrating antithrombotic activity [126, 127, 128, 129]. Importantly, different forms of vitamin E display distinct biological activities. For instance, mixed tocopherols (containing α‐, γ‐ and δ‐forms) are more effective than pure α‐tocopherol in inhibiting platelet aggregation and intracellular lipid peroxidation [130, 131]. This phenomenon is reminiscent of the activity differences observed among vitamin A isomers, highlighting the significant influence of chemical structure on vitamin function. Although preclinical studies have demonstrated clear protective effects, evidence from large‐scale clinical trials remains inconsistent. Some studies suggest that vitamin E supplementation may reduce cardiovascular risk [132]. However, evidence from high‐quality randomised controlled trials does not support the purported cardiovascular protective effects of vitamin E. The WAVE study, which enrolled 423 postmenopausal women with coronary heart disease receiving 400 IU of vitamin E plus 500 mg of vitamin C twice daily, showed no significant benefit in slowing the progression of coronary artery stenosis [133]. On the contrary, it indicated a potential increase in adverse outcomes such as death or myocardial infarction. Similarly, the HOPE and HOPE‐TOO trials, involving 9541 high‐risk patients with a median follow‐up of 7 years, demonstrated that long‐term daily supplementation with 400 IU of vitamin E did not reduce the incidence of cardiovascular events or cancer but was associated with a higher risk of heart failure and related hospitalizations [134]. These neutral or even harmful findings from large‐scale RCTs sharply contrast with the benefits suggested by earlier observational studies, underscoring the limited strength of evidence supporting vitamin E in CVD prevention and the need for cautious interpretation. This pattern mirrors findings for other antioxidant nutrients, implying that antioxidant interventions alone may be insufficient to address the complexity of human AS. Consequently, current evidence does not support routine high‐dose vitamin E supplementation in the general population or in patients with CVD.

#### 4.1.6 Vitamin K

Vitamin K is a class of fat‐soluble vitamins with phylloquinone bioactivity, primarily including phylloquinone (K1) from plant sources and menaquinone (K2) produced by bacterial fermentation [135]. Beyond its classical role in coagulation, vitamin K has gained increasing attention for its involvement in AS. Vitamin K activates multiple vitamin K‐dependent proteins through carboxylation, among which matrix Gla protein (MGP) serves as a key inhibitor of vascular calcification. Activated MGP effectively suppresses calcification in vascular smooth muscle cells, thereby slowing AS progression [136, 137]. Notably, unlike vitamin D, which primarily regulates systemic calcium‐phosphorus homeostasis, vitamin K acts locally within tissues to directly inhibit aberrant calcium deposition. Conversely, vitamin K antagonists (e.g., warfarin) inhibit MGP activation, exacerbating vascular calcification, accelerating atherosclerotic progression and potentially increasing hypercoagulability, thereby promoting thrombosis [138]. Moreover, synergistic effects have been observed between vitamin K and certain trace elements. For instance, combined supplementation of vitamin K and selenium has been shown to improve serum lipid profiles by lowering total cholesterol, triglycerides and LDL levels while increasing HDL, exerting anti‐atherosclerotic effects [139]. Most observational clinical studies support an association between higher vitamin K intake and lower cardiovascular risk [140, 141]. However, randomised controlled trials have not consistently demonstrated that vitamin K supplementation can sustainably delay vascular calcification, AS or arterial stiffness. Existing studies exhibit significant heterogeneity and limitations in sample size, follow‐up duration and risk of bias. Although some subgroup analyses (e.g., in individuals with preexisting calcification or high adherence) suggest potential benefits, this finding has not been universally confirmed in primary analyses [142]. In summary, although vitamin K has a well‐defined molecular mechanism for activating MGP and inhibiting vascular calcification, its clinical efficacy requires further validation. Future research should aim to identify subpopulations most likely to benefit from vitamin K supplementation and explore its synergistic effects with other nutrients (e.g., vitamin D, selenium) to develop more targeted intervention strategies.

### 4.2 Minerals and AS

#### 4.2.1 Copper

Copper is an essential trace element whose homeostasis is tightly regulated. Studies have shown that both excess and deficiency of copper can lead to pathological changes associated with AS [143]. By analysing transition metal ions in human atherosclerotic plaques, researchers have found elevated copper levels in carotid artery lesions [144]. Additionally, serum copper levels have been negatively correlated with cIMT [145]. A multicentre European study found that increased dietary copper enhances cellular antioxidant capacity, which may help prevent vascular damage [146]. In a high‐fat diet (HFD) rabbit model, Lamb et al. [147] discovered that both copper deficiency and excess in the diet increased the susceptibility to aortic AS. The mechanisms by which copper deficiency or excess promotes AS may involve increased oxidative stress, endothelial dysfunction and disrupted lipid metabolism [148, 149].

Changes in copper levels can lead to the production of ROS [150]. Elevated levels of free copper ions can generate more hydroxyl radicals through the Fenton reaction, increasing oxidative stress. Increased copper levels can promote lipid peroxidation, leading to the oxidation of LDL particles, forming ox‐LDL, a key factor in atherosclerotic plaque formation [151]. Animal studies have shown that copper overload‐induced lipid peroxidation can decrease superoxide dismutase (SOD) activity and glutathione levels in rat brain tissue, thereby increasing oxidative stress [152]. Moreover, copper deficiency may also be a risk factor for AS. Copper is a vital cofactor in various redox reactions within the body, and its deficiency can reduce Cu/Zn‐SOD activity and cause complex I oxidation inactivation, leading to increased ROS production in copper‐deficient cells and exacerbating oxidative stress [153]. ECs damage is considered one of the initiating factors of AS. Zhong et al. [154] found that excess copper increases oxidative stress, leading to ECs dysfunction. Hcy levels are an important risk factor for AS, and copper can catalyse Hcy oxidation. Further research revealed that incubation of Hcy with copper for 4 h resulted in ECs damage [155]. Copper is also involved in the synthesis of fatty acids and cholesterol, as well as various forms of lipoprotein metabolism, but the relationship between copper and lipid metabolism remains controversial. Xu et al. [156] using multiple linear regression and logistic regression models, found that TC and LDL levels in women increased with rising copper concentrations. A Mendelian study found that higher blood levels of iron and copper were protective factors against dyslipidemia, hyperlipidemia and hypercholesterolemia [157]. However, a meta‐analysis of randomised controlled trials found that copper supplementation did not significantly affect blood lipids. Specifically, compared to the control group, there were no significant differences in TC, LDL and HDL levels in patients who received copper supplementation [158]. These conflicting results may be due to differences in the populations studied, the doses of copper supplementation and the duration of the studies. Future high‐quality research is needed to investigate the specific mechanisms by which copper affects AS and to determine the optimal supplementation dose. This will help develop more precise and effective prevention and treatment strategies.

#### 4.2.2 Zinc

Zinc plays multifaceted roles in maintaining immune homeostasis and vascular health, with its primary mechanisms mediated through antioxidant and anti‐inflammatory pathways. At the molecular level, zinc markedly suppresses NF‐κB activation by modulating the A20 and PPAR signalling pathways. Zinc deficiency decreases the expression of PPARα and PPARγ in endothelial cells, thereby enhancing NF‐κB DNA‐binding activity and upregulating proinflammatory cytokines, such as IL‐1β, IL‐6 and TNF‐α [159, 160, 161]. Unlike vitamin D, which predominantly regulates inflammatory gene transcription via the VDR nuclear receptor, zinc more commonly influences inflammatory pathways such as NF‐κB indirectly through transcriptional regulators, including zinc finger proteins (e.g., A20, ZEB2). Recent studies have demonstrated that the zinc finger protein ZEB2 inhibits NF‐κB pathway activation by suppressing the phosphorylation of p65 and IκBα, consequently reducing inflammatory cytokine production [162]. Moreover, zinc concentrations in human endothelial cells exhibit a significant positive correlation with A20 protein expression, further underscoring zinc's pivotal role in regulating inflammation [163]. In antioxidant defence, zinc serves as a structural cofactor for copper/zinc superoxide dismutase (Cu/Zn‐SOD), which is essential for maintaining the enzyme's stability and activity. Zinc deficiency induces conformational alterations in the SOD1 protein, thereby reducing its activity and aggravating oxidative stress [164]. This mechanism contrasts with that of copper: although both are indispensable cofactors for SOD1, zinc primarily stabilises the enzyme structure, whereas copper directly participates in catalytic reactions. Excessive oxidative stress can react with NO to generate peroxynitrite, which not only decreases NO bioavailability but also impairs vascular endothelial function [165]. Zinc supplementation has been shown to mitigate oxidative stress, protect vascular smooth muscle cells and promote vasodilation [166]. In lipid metabolism, zinc regulates the expression of key reverse cholesterol transport proteins, such as ABCA1 and ApoE, through the zinc finger protein ZNF580, while simultaneously suppressing the expression of CD36 and PPAR‐γ. These effects collectively reduce lipid uptake and inhibit foam cell formation [167]. Meta‐analyses have shown that zinc supplementation in humans significantly improves lipid profiles by lowering total cholesterol, LDL‐C, and triglyceride levels, while increasing HDL‐C [168, 169]. Consistently, animal studies demonstrate that zinc deficiency exacerbates atherosclerotic lesions in apoE^−^/^−^ mice, whereas zinc supplementation markedly improves lipid levels and reduces aortic lesion areas [170, 171]. Although mechanistic studies demonstrate clear protective effects, findings from population‐based studies remain inconsistent. A systematic review of prospective cohort studies indicated that serum zinc levels show no significant association with CVD risk in the general (non‐clinical) population. Notably, higher serum zinc levels demonstrated a significant protective effect in vulnerable populations with existing type 2 diabetes or undergoing coronary angiography [172]. In contrast, higher dietary zinc intake was inversely associated with vascular mortality risk in elderly cohorts [173]. These discrepancies suggest that zinc's protective effects may depend on age, baseline zinc status or other population characteristics, underscoring the need for validation in larger‐scale clinical trials.

#### 4.2.3 Iron

Iron plays a complex and critical role in the development and progression of AS [174, 175]. In the 1980s, Sullivan proposed the ‘iron hypothesis’, suggesting that hyperferric states promote CVD by catalysing oxidative reactions, whereas iron deficiency may exert protective effects. Subsequent studies, however, have revealed more intricate mechanisms. Iron overload results in substantial vascular iron accumulation, which is strongly associated with plaque formation, increased oxidative stress and vascular dysfunction. Through the Fenton reaction, iron catalyses the production of ROS, leading to abnormal lipid distribution, increased vascular permeability, sustained endothelial activation, release of proinflammatory mediators and reduced NO bioavailability—processes that collectively accelerate AS progression [176, 177]. Moreover, excessive iron intake may promote the formation of advanced glycation end‐products (AGEs) and accelerate oxidative modification of lipoproteins [178]. It is noteworthy that iron deficiency also adversely affects the progression of AS. Studies indicate that reduced iron levels are closely associated with elevated levels of inflammatory mediators such as TNF‐α, IL‐6 and C‐reactive protein (CRP), which promote the formation of atherosclerotic plaques [179]. A macrophage‐specific ferroportin (Fpn1) knockout mouse model demonstrated that iron accumulation within macrophages inhibits the LXRα–ABCA1/ABCG1 cholesterol efflux pathway, thereby promoting foam cell formation and accelerating AS progression [180]. Further studies have shown that a high‐iron diet markedly increases macrophage infiltration within plaques, enhances the release of proinflammatory cytokines such as TNF‐α, IL‐6 and IL‐1β and drives macrophage polarisation toward the M1 proinflammatory phenotype, collectively exacerbating the pathological progression of AS [181]. Imbalances in iron metabolism can also trigger ferroptosis, a form of iron‐dependent regulated cell death characterised by the excessive accumulation of lipid peroxides [182]. Elevated intracellular free iron generates large amounts of ROS via the Fenton reaction, promoting polyunsaturated fatty acid (PUFA) peroxidation and directly inducing ferroptosis [183]. Treatment with the ferroptosis inhibitor Ferrostatin‐1 (Fer‐1) upregulates SLC7A11/GPX4 expression, suppresses lipid peroxidation and improves endothelial function, thereby markedly attenuating AS lesions [184]. Iron chelation therapy has been shown to improve endothelium‐dependent vasodilation and slow the progression of coronary AS [185]. Interestingly, a randomised controlled trial in patients with peripheral artery disease demonstrated that reducing iron stores via phlebotomy did not significantly lower the risk of all‐cause mortality or composite cardiovascular endpoints [186]. Although this multicentre study was rigorously designed, the final results were negative, and the potential benefits suggested by post hoc subgroup analyses (e.g., in younger patients) require further validation. Recent Mendelian randomisation studies similarly suggest that genetically determined hyperferritinemia may even be associated with lower coronary AS risk [187], highlighting the complex and potentially contradictory role of iron in AS. Current evidence does not allow definitive conclusions regarding a causal relationship between iron storage levels and cardiovascular risk. Future studies are warranted to further clarify the complex role of iron homeostasis in AS and to explore precision therapeutic strategies targeting iron metabolism.

#### 4.2.4 Selenium

Selenium is an essential dietary micronutrient required for maintaining normal physiological functions in humans. It predominantly exists in the form of selenoproteins and plays a central role in antioxidant defence and immune regulation [188]. Epidemiological studies have shown that combined supplementation with selenium and coenzyme Q10 significantly reduces cardiovascular mortality in elderly Swedish populations, with protective effects persisting for up to 10 years after the intervention [189, 190]. Selenium exerts anti‐atherosclerotic effects through multiple molecular mechanisms. As a key component of glutathione peroxidases (GPx), it catalyses the reduction of hydrogen peroxide and lipid hydroperoxides, thereby effectively protecting cell membranes and LDL from oxidative damage [191, 192]. Unlike direct antioxidants such as vitamins C and E, selenium provides sustained antioxidant protection via enzymatic activity. Experimental studies have further demonstrated that selenium supplementation significantly enhances GPx‐1 activity in spontaneously hypertensive rats, reduces lipid peroxidation and lowers levels of AGEs [47]. Additionally, selenium exerts a dose‐dependent inhibitory effect on TNF‐α‐mediated expression of endothelial adhesion molecules (intercellular adhesion molecule‐1 (ICAM‐1), VCAM‐1, E‐selectin), thereby reducing monocyte adhesion to the endothelium. It also suppresses the release of proinflammatory cytokines such as TNF‐α and IL‐6, potentially acting synergistically with zinc in mitigating vascular inflammation [193, 194, 195]. In terms of vascular cell protection and regulation of calcification, selenium modulates the PI3K/AKT pathway via molecules such as selenoprotein S, thereby inhibiting oxidative stress and endoplasmic reticulum stress‐induced apoptosis in VSMCs [196, 197]. In addition, selenium upregulates osteoprotegerin expression, suppresses the activity of receptor activator of NF‐κB ligand (RANKL), and reduces the differentiation of vascular smooth muscle cells into osteoblast‐like cells, thereby inhibiting vascular calcification [198]. Moreover, selenium can modulate the levels of the anti‐apoptotic protein Bcl‐2 and the expression of the autophagy‐related protein LC3‐II, thereby promoting autophagy and maintaining vascular cell homeostasis [199, 200]. It is noteworthy that selenium supplementation provides significant benefits primarily in individuals with low selenium status while having minimal effects in those with adequate selenium levels [201]. This pattern is similar to the mechanisms observed for other nutrients, such as zinc and vitamin D, highlighting the critical role of baseline nutritional status in determining intervention outcomes. However, unfortunately, randomised controlled trials investigating the relationship between selenium levels and AS are lacking. Therefore, further research is needed to elucidate the complex interaction between selenium levels and AS.

#### 4.2.5 Magnesium

Magnesium is the fourth most abundant cation in the human body, and its deficiency has become a significant public health issue among adults [202]. Cross‐sectional data from southern Germany indicate a significant association between serum magnesium levels and carotid plaque [203]. In the regulation of vascular function, magnesium serves as a natural antagonist to calcium. It lowers intracellular calcium levels by inhibiting inositol trisphosphate (IP₃)‐mediated calcium release from the sarcoplasmic reticulum and by suppressing calcium ATPase activity, thereby promoting vasodilation [204]. In contrast to potassium, whose vasodilatory effects are mediated by membrane hyperpolarisation, magnesium primarily acts through direct antagonism of calcium signalling pathways. Additionally, magnesium enhances the production of vasodilators, including prostacyclin and nitric oxide, while reducing vascular responsiveness to angiotensin II and catecholamines [204, 205]. A large cross‐sectional study based on NHANES data reported a significant association between high dietary magnesium intake and lower prevalence of hypertension, a finding that has been confirmed in European and Asian populations [206, 207, 208, 209]. Regarding its anti‐inflammatory effects, magnesium sulphate inhibits activation of the NF‐κB signalling pathway by suppressing IκBα degradation and p65 nuclear translocation, thereby reducing the expression of interleukin‐8 and ICAM‐1 in human umbilical vein endothelial cells [210]. Prospective studies have demonstrated a negative correlation between magnesium intake and circulating levels of high‐sensitivity C‐reactive protein (hs‐CRP), interleukin‐6 and fibrinogen [211]. Cellular and animal studies further indicate that magnesium deficiency induces leukocyte and macrophage activation, promotes the release of inflammatory cytokines and acute phase proteins, and increases free radical generation. Conversely, elevating extracellular magnesium concentrations attenuates inflammatory cell infiltration and dampens inflammatory responses [212, 213, 214]. Magnesium deficiency enhances the production of free radicals by neutrophils and macrophages, increases lipid peroxidation, leading to ECs damage and AS [215]. In terms of metabolic regulation, low magnesium levels may impair glucose homeostasis, increase insulin resistance and elevate the risk of hyperglycemia. Randomised controlled trials have shown that oral magnesium supplementation significantly improves HOMA‐IR, fasting blood glucose and triglyceride levels [216]. Meta‐analyses further indicate that patients with lower serum magnesium levels exhibit higher triglycerides, total cholesterol and low‐density lipoprotein cholesterol, along with reduced high‐density lipoprotein cholesterol [217]. In animal models, dietary magnesium supplementation markedly ameliorated vascular dysfunction in rats with metabolic syndrome and chronic kidney disease. The underlying mechanisms include reduced lipid peroxidation, downregulation of aortic IL‐1β and IL‐6 expression, enhanced nitric oxide bioavailability and decreased endothelin‐1 levels [218]. This multifaceted protective mechanism differs from selenium's antioxidant effects mediated via the GPx enzyme system, highlighting the unique role of magnesium in cardiovascular protection. A systematic review and meta‐analysis of randomised controlled trials concluded that magnesium supplementation significantly improves flow‐mediated dilation in adults, though it demonstrated no overall effect on carotid intima‐media thickness. However, these findings are constrained by the small sample sizes and considerable heterogeneity among the included trials [219]. In contrast, a large prospective cohort study and an updated meta‐analysis reported an inverse association between serum magnesium levels and coronary artery disease risk, which appeared more pronounced in women [220]. While the latter provides more robust epidemiological support, its observational nature precludes causal inference. Collectively, the available evidence remains inadequate to justify routine magnesium supplementation in the general population for cardiovascular prevention. Further clarification of its potential benefit, particularly in high‐risk subgroups, will require large, well‐designed RCTs.

#### 4.2.6 Calcium and Phosphorus

Calcium is a vital element in human physiological and biochemical processes. Apart from its role as a major constituent of bones and teeth, calcium participates in numerous molecular pathways, including nerve excitability, muscle contraction, hormone action, enzyme secretion, cell motility and blood clotting [221, 222]. Additionally, calcium plays a significant role in signal transduction pathways, acting as a second messenger in various cells [223]. Several studies have demonstrated that calcium influences AS development by inducing vascular calcification, activating inflammatory pathways and compromising endothelial function, with these effects being dose‐dependent and varying according to individual nutritional status [224, 225, 226]. Moreover, subtle increases in cIMT have been observed in patients with primary hyperparathyroidism and elevated serum calcium levels [227]. Furthermore, calcium antagonists have long been recognised for their anti‐atherosclerotic properties [228]. However, some studies have indicated that hypercalcemia leads to calcification of atherosclerotic plaques. Calcified atherosclerotic plaques are biomechanically more stable, less prone to rupture and less likely to produce symptoms compared to non‐calcified carotid plaques [229, 230]. Therefore, the involvement of calcium in atherosclerotic progression continues to be debated, requiring additional studies for clarification.

Phosphorus is an essential element for maintaining normal physiological functions in the human body. Not only does it work with calcium to support the formation of bones and teeth, but it is also a crucial component of ATP, DNA and RNA [231, 232]. Adequate levels of phosphorus play a vital role in cellular signalling pathways. In the context of AS, the impact of phosphorus largely depends on maintaining balanced levels within the body. Current research widely acknowledges that hyperphosphatemia significantly increases the risk of AS [233, 234]. Elevated blood phosphorus levels are associated with coronary AS development even within the normal range [235]. In individuals with metabolic syndrome, particularly those with uremia, disturbances in phosphorus metabolism are more likely to lead to vascular calcification and AS. Additionally, early management of hyperphosphatemia in dialysis patients substantially reduces the risk of AS and its calcification [236, 237]. It is important to note that the specific pathophysiological mechanisms linking phosphorus to AS remain poorly understood and warrant further investigation.

#### 4.2.7 Potassium

Potassium is the major intracellular cation, playing a key role in maintaining cellular electrochemical balance and regulating blood pressure [238]. Adequate potassium intake confers multifaceted protective effects against AS by promoting sodium excretion and exerting direct vascular actions, including inhibition of vascular smooth muscle cell migration, enhancement of endothelial function and prevention of vascular calcification [239]. Regarding the inhibition of VSMC migration, studies have shown that potassium markedly attenuates platelet‐derived growth factor (PDGF‐BB)‐induced VSMC migration under varying extracellular potassium concentrations [240, 241, 242]. In contrast to magnesium, which primarily acts through antagonism of calcium signalling pathways, potassium modulates cell migration by regulating membrane potential and ionic gradients. In vivo studies further demonstrate that increased dietary potassium intake reduces neointimal formation in a rat carotid balloon injury model, likely by inhibiting VSMC proliferation and migration [243]. Studies have shown that mice fed a low‐potassium diet (0.3%) exhibit accelerated vascular calcification and increased aortic stiffness compared with mice receiving normal potassium intake [244]. Mechanistic investigations indicate that reduced extracellular potassium enhances intracellular calcium influx, which activates the cAMP response element‐binding protein (CREB) signalling pathway [245]. Additionally, dietary potassium deficiency induces cell membrane depolarisation through activation of inward rectifier potassium channels, leading to the opening of voltage‐gated calcium channels and sustained intracellular calcium elevation. The increased intracellular calcium further activates the CREB pathway, enhancing autophagy and ultimately promoting the differentiation of VSMCs into osteoblast‐like cells, thereby accelerating vascular calcification [244]. Furthermore, two cross‐sectional studies conducted in Chinese populations reported a positive association between elevated urinary sodium‐to‐potassium ratios and carotid AS, highlighting the critical role of potassium‐sodium balance in AS prevention [246, 247]. Taken together, potassium exerts anti‐atherosclerotic effects through multiple mechanisms, including inhibition of VSMC migration, regulation of vascular calcification and maintenance of electrolyte homeostasis. Its actions show synergistic interactions with divalent cations such as magnesium, while also operating through distinct ion channel and signalling pathway‐mediated mechanisms. Future studies should further investigate the network of interactions between potassium and other minerals to provide a scientific basis for the development of evidence‐based dietary guidelines.

## 5 Application and Practice of Micronutrients in the Prevention and Treatment of AS

### 5.1 Current Status and Challenges of Micronutrient Intervention Strategies

Current evidence synthesis reveals that most fundamental research has predominantly focused on investigating the effects of individual micronutrients on AS, while largely overlooking other critical factors such as interactions between micronutrients and individual variability. Consequently, there exists a significant disparity between theoretical findings and clinical outcomes. Through meta‐analyses, Jenkins et al. [248] demonstrated that systematic evaluations of large‐scale randomised controlled trials indicate common vitamin and mineral supplements (e.g., multivitamins, vitamin D, calcium, vitamin C) show no significant effects on CVD prevention or all‐cause mortality. Similarly, Sunkara et al. [249] reported that although micronutrients theoretically possess the potential to modulate oxidative stress and inflammation, large‐scale clinical trials consistently demonstrate that micronutrient supplementation fails to improve cardiovascular outcomes. Certain interventions (e.g., β‐carotene) may even pose risks to high‐risk subgroups (e.g., smokers) [250]. This inconsistency further underscores the limitations of current research paradigms: studying isolated micronutrients without considering overall dietary context ignores critical nutrient‐nutrient synergies and antagonisms, while inadequate attention to baseline nutritional status and population heterogeneity further compounds these issues. These conclusions collectively indicate that existing evidence does not support the widespread use of micronutrient supplements in the general population. Furthermore, the U.S. Preventive Services Task Force (USPSTF) explicitly recommends against the routine use of vitamin/mineral supplements for CVD prevention [251]. This raises a pivotal question: How should micronutrients be properly understood and utilised?

While clinical data indicate that most micronutrient supplements show limited efficacy in preventing or treating CVD, notable exceptions exist. The CSPPT (China Stroke Primary Prevention Trial) demonstrated that folic acid supplementation reduced stroke risk by 21% in hypertensive patients, highlighting its preventive potential [252]. However, this benefit was observed exclusively in regions without folic acid fortification policies (e.g., China). In contrast, no significant effect was seen in areas with adequate dietary folate intake (e.g., the United States and Canada). These findings prompt a crucial question: Could precision nutrition strategies—tailored to individual nutritional status, genetic background and other factors—optimise supplementation approaches to more effectively mitigate CVD risk? Importantly, lacking supplemental benefits in regions with folate fortification underscores a fundamental principle: a well‐balanced diet can sufficiently meet folate requirements. This observation reinforces that obtaining micronutrients through diversified whole foods, rather than indiscriminate supplement use, should be the primary strategy for preventing AS and other CVDs. Targeted supplementation should be reserved only for specific populations with clinically confirmed nutrient deficiencies or elevated metabolic demands.

### 5.2 Nutritional and Dietary Recommendations

Micronutrients are essential in the human body and involve various biological processes. Micronutrients in the human body are mainly dependent on daily dietary intake (Table 1). A diverse diet ensures a comprehensive intake of various micronutrients and minimises the occurrence of diseases related to micronutrient deficiencies. Epidemiological studies across diverse populations have confirmed a positive correlation between Dietary Diversity Score (DDS) and micronutrient levels in the body. When DDS reaches 6 or above, the risk of micronutrient deficiency can be reduced by 58%, while also slowing carotid intima‐media thickness progression by 23% [253, 254, 255]. This protective effect mainly stems from the synergistic interactions of various antioxidant vitamins (such as vitamins C and E) and balanced regulation among minerals. The dietary patterns that have garnered the most respect in the current scientific community are the Mediterranean Diet [256] and the DASH Diet [257], both of which emphasise the intake of natural foods, dietary fibre and high‐quality fats to improve the absorption and utilisation of micronutrients, and effectively alleviate the progression of chronic diseases such as CVD and promote health [258]. In a cross‐sectional observational study of breast cancer patients, Negrati et al. [259] found that adherence to a Mediterranean diet increased the levels of fat‐soluble vitamins in breast cancer patients and helped improve cardiometabolic parameters. It is important to note that optimising diets based on factors such as physical condition, lifestyle, age, gender and medical history, while ensuring adequate and appropriate micronutrient intake, can facilitate the development of personalised nutritional plans. Such tailored approaches better address health needs and contribute to disease prevention. Adopting a personalised approach ensures that dietary recommendations are aligned with an individual's or a population's specific health status, thereby maximising potential health benefits while minimising the risk of adverse effects.

**TABLE 1:** Primary dietary sources, recommended intakes and clinical manifestations of micronutrient deficiency.

Micronutrients	Mainsources	Optimal intake for healthy adults (adjusted for different situations)	Effects of deficiency and/or overload on cardiovascular health
Vitamin A	Animal products (liver, kidney, oil, dairy products and eggs) and plants (red and orange vegetables)	About 2600 IU/day, not more than 5000 IU/day	Eye symptoms: night blindness, xerophthalmia Skin symptoms: Dry and rough Impaired immune function, Reproductive system dysfunction
B Vitamins	Cereal: B1, B2, B5, B6, B9 Legume: B1, B3, B9 Vegetables: B2, B6 Animal liver: B3, B5, B9, B12	Not less than the following intake daily B1: 1.1–1.2 mg, B2: 1.0–1.3 mg, B3: 11–12 mg, B5: 5 mg, B6: 1.3–1.7 mg, B9: 400 μg B12: 2.4 μg	Oral ulcers, anaemia, skin abnormalities, gastrointestinal discomfort, neurological symptoms
Vitamin C	Citrus fruits, strawberries, tomatoes, potatoes and green leafy vegetables	40–120 mg/day	Anorexia, weakness, bleeding symptoms (gums, skin, digestive tract, etc.), limb pain
Vitamin D	Animal food: D3 Plants and yeast: D2	400–600 IU/day	Bone symptoms (bone pain, bone deformity, osteoporosis, dysplasia, etc.) Muscle symptoms (muscle weakness, muscle spasm, etc.) Psychiatric symptoms (depression, insomnia, etc.) Blood calcium and phosphorus levels decreased
Vitamin E	Oils, cereals, plants, legumes, animal‐derived products	15 mg/day (22.4 or 33.3 IU of natural or synthetic α‐tocopherol)	Neurodegenerative diseases (dysphagia, slurred speech, ataxia), hemolytic anaemia, retinopathy
Vitamin K	Cheese, yolk, cyanobacteria, algae and green plants (broccoli, brussels sprouts, cabbage, kale, etc.)	1 mg/kg per day	Bleeding symptoms (mucocutaneous bleeding, gastrointestinal bleeding, visceral bleeding, neonatal intracranial haemorrhage, etc.)
Copper	Nuts, poultry, animal livers, legumes, grains and seeds	2.10–3.00 mg/day	Anaemia, decreased resistance, osteoporosis, dyslipidemia, psychobehavioral abnormality, growth retardation
Zinc	Red meat, poultry, shellfish, legumes, dairy products, nuts and seeds	8.5–12.5 mg/day	Digestive symptoms (anorexia, pica), growth retardation, skin problems (dermatitis, mouth ulcers, alopecia), slow wound healing
Iron	Animal foods: red meat, poultry, liver, seafood Plant foods: vegetables, legumes, grains, nuts	10–15 mg/day, not more than 40 mg/day	Fatigue, Dizziness, Palpitations, Skin problems (abnormal pallor, concave nails), Poor breathing
Selenium	Cereals, meat, eggs, dairy products, fishes, seafood, milk and nuts	55 μg/day	Dark yellow skin, endocrine disorders (diarrhoea, premature ovarian failure), circulatory system symptoms (chest tightness, palpitation, heart failure), nervous system symptoms (insomnia, irritability)
Magnesium	Nuts, legumes, cereals, chocolate, seafood and leafy green vegetables	330 mg/day	Neuromuscular symptoms (muscle tremor, hand‐foot twitching), Cardiovascular symptom (arrhythmia), Digestive symptoms (loss of appetite, nausea, vomiting)
Calcium	Dairy products, legumes and soy products, green leafy vegetables, nuts, fishes	800 mg/day	Muscle spasms and twitches, arrhythmias, osteoporosis, rickets
Phosphorus	Meat, fishes, dairy products, cereals, legumes	700–1000 mg/day	Digestive symptoms (loss of appetite, nausea, vomiting) Skeletal symptoms (muscle pain, muscle weakness) Neurologic symptoms (dizziness, headache, drowsiness) Cardiovascular symptoms (low blood pressure, heart failure)
Potassium	Fruits (bananas, oranges, strawberries, kiwifruit, etc.), vegetables (potatoes, spinach, kale, cauliflower, etc.), dairy products, fungi	2000 mg/day	General weakness, shortness of breath, loss of appetite, abdominal distension, vomiting and cardiac arrhythmias

### 5.3 The Use of Micronutrient Supplements

In daily life, the use of micronutrient supplements should be approached with caution. First and foremost, a proper evaluation of individuals should be conducted. Micronutrient supplementation is not limited to individuals with clinically diagnosed deficiencies. For populations with elevated potential requirements, such as those who are pregnant, elderly, affected by chronic diseases or undergoing long‐term medication, appropriate supplementation should be considered based on careful assessment, even when haematological indicators remain within normal ranges [260]. Statistical analysis indicates that micronutrient deficiencies affect approximately 2 billion individuals globally, with infants, young children, school‐age children, pregnant women, athletes, the elderly, and those with impaired absorption or long‐term medication use being particularly vulnerable. Infants and young children are particularly vulnerable to iron deficiency due to their high demand for iron in rapid growth and development, and thus require regular iron supplementation [261]. School‐age children often demonstrate deficiencies in zinc, vitamin D and iodine, necessitating targeted interventions through dietary modifications or supplements [262]. Pregnant women should prioritise the supplementation of essential micronutrients such as folic acid, iron and calcium to meet the demands of foetal growth and development [263]. In the case of patients suffering from CVD, such as AS, it is recommended that blood micronutrient levels be tested regularly due to the increased consumption of micronutrients caused by the chronic inflammatory state and supplementation programmes should be formulated based on the results [264]. Such patients should give priority to dietary supplementation, and if dietary intake is insufficient, individual conditions, drug interactions and other factors should be taken into account for safety assessment and selection of personalised single or combination supplements. Scientific and rational micronutrient supplementation is not only effective in preventing and treating deficiencies but also in supporting specific physiological states and ameliorating chronic diseases, thereby enhancing overall health.

Scientific and rational micronutrient supplementation must strictly follow the ‘U‐shaped curve’ principle, as both deficiency and excess can be detrimental to health. Thus, nutritional strategies should comprehensively consider individual requirements, appropriate dosages and nutrient bioavailability. With respect to minerals, abnormal magnesium levels may lead to multisystem dysfunction, including muscle weakness, arrhythmias, and, in severe cases, respiratory failure [215]. Excessive zinc intake (> 40 mg/day) can interfere with copper absorption and metabolism, resulting in anaemia [265]. Serum potassium levels above 6.3 mM increase the risk of arrhythmias and even cardiac arrest [266]. Iron overload is particularly hazardous and may lead to life‐threatening conditions such as acute liver failure [267]. Vitamin supplementation also requires careful monitoring due to potential risks. Fat‐soluble vitamins (such as A, D, E and K) can accumulate in the body and lead to toxic effects. For example, excessive vitamin A intake has been linked to liver damage and increased intracranial pressure [268]; chronic vitamin D overdose may result in hypercalcemia, vascular calcification and kidney injury [269]; and excessive vitamin E can interfere with normal blood‐clotting mechanisms [270]. Although water‐soluble vitamins are generally not stored in significant amounts, extremely high doses (e.g., vitamin B6) may still cause adverse effects such as peripheral neuropathy [271]. Two recent case studies demonstrate the severe consequences of excessive fat‐soluble vitamin supplementation. In the United States, Alchalabi et al. [272] reported a case of a 69‐year‐old female breast cancer patient who consumed vitamin A supplements exceeding conventional doses (25,000 IU/day) daily for several months, ultimately developing hypercalcemia due to prolonged overdose. Additionally, in the United Kingdom, Khan and colleagues reported a 76‐year‐old male who developed severe hypercalcemia (serum 25(OH)D > 250 nmol/L) and acute renal failure due to prolonged intake of high‐dose vitamin D supplements (10,000–20,000 IU/day, increased to 50,000 IU/day when experiencing generalised weakness and dizziness) [273]. While the body can partially mitigate overconsumption through mechanisms such as renal excretion and biotransformation, exceeding individual tolerance thresholds may ultimately result in multi‐organ toxicity.

When evaluating micronutrient supplementation, bioavailability is a critical consideration. Absorption rates differ substantially among various compound forms. Organic forms, such as magnesium citrate, ferrous glycinate, methylfolate and natural vitamin E (d‐α‐tocopherol), generally demonstrate higher bioavailability than inorganic salts (e.g., magnesium oxide, ferrous sulphate) or synthetic forms (e.g., dl‐α‐tocopherol) [274, 275, 276]. Interactions between nutrients further influence bioavailability. For instance, vitamin C reduces ferric iron to the more absorbable ferrous form and forms soluble complexes that enhance absorption [277], while vitamin D promotes intestinal calcium absorption by upregulating calcium‐binding protein expression via gene regulation [278]. In contrast, competitive inhibition can occur: excessive zinc supplementation impairs copper uptake by inhibiting copper transporters [279], and high‐dose calcium can reduce the absorption of other divalent minerals such as zinc and iron through competition for shared ion channels [280]. The gut microbiota also plays an essential role in modulating bioavailability. Certain bacterial populations, including *Lactobacillus* and *Bifidobacterium*, synthesise vitamin K₂ and several B vitamins [281]. In addition, their metabolic products, short‐chain fatty acids, lower intestinal pH, and thereby facilitate the dissolution and absorption of minerals such as calcium, magnesium and iron [282]. Moreover, the composition and function of microbial communities directly affect the integrity of the intestinal mucosal barrier and the expression of nutrient transporters [283]. Overall, micronutrient supplementation should adhere to the principles of individualization and appropriate dosage, aiming to avoid both the health risks associated with deficiency and the toxic reactions caused by excess. Therefore, in practical application, strict adherence to safe intake standards is essential, and personalised supplementation strategies must be developed based on an individual's physiological state, nutritional status and health requirements.

### 5.4 Micronutrient Combined With Drug Therapy

In contemporary clinical practice, there is an increasing utilisation of diverse pharmacological agents, concomitant with a rise in adverse drug reactions. Long‐term drug users, particularly the elderly, frequently exhibit micronutrient malabsorption, which can result in chronic micronutrient deficiencies. These deficiencies can further precipitate metabolic disorders, engendering severe health complications. It is well established that drugs and micronutrients are absorbed and metabolised via the same transport and metabolic pathways in the human body. Therefore, interactions between them are inevitable [284]. The intake of drugs can affect the absorption of micronutrients, and similarly, micronutrients may adversely affect the action of drugs.

Statins, the core lipid‐lowering agents in the management of CVD, have been shown to significantly reduce LDL‐C levels by inhibiting 3‐hydroxy‐3‐methylglutaryl coenzyme A (HMG‐CoA) reductase [285]. However, prolonged use of statins has been associated with several potential adverse effects, including decreased coenzyme Q10 synthesis, abnormal vitamin D metabolism and increased selenium depletion. These factors, in turn, may trigger a range of symptoms, including muscle symptoms, fatigue and oxidative stress [286]. Studies have shown that statin combined supplementation with coenzyme Q10 and magnesium is effective in improving muscle function [287, 288], while supplementation with vitamin D and vitamin B12 enhances anti‐inflammatory effects and further reduces the risk of cardiovascular events [289, 290]. Therefore, rational micronutrient supplementation not only alleviates the side effects of statins but also synergistically enhances their therapeutic effects, providing important support for the comprehensive management of CVD. Metformin is a commonly used hypoglycaemic agent. In addition to its role in glycaemic control, it improves endothelial dysfunction, reduces oxidative stress and lipid levels and protects cardiovascular health [291, 292]. The results of a randomised controlled trial showed that serum vitamin B12 concentrations were significantly reduced in a dose‐dependent manner in patients treated with metformin [293]. In addition, diabetes is often associated with elevated Hcy levels, which can increase the risk of CVD [294]. Therefore, supplementation with vitamin B12 and folic acid in metformin users can mitigate the adverse effects of metformin and enhance the glucose‐lowering effect and cardiovascular protection [295]. Micronutrient monitoring and intervention should be intensified in long‐term metformin users to optimise therapeutic efficacy and enhance the quality of life for patients. Thus, in routine medication practices, more attention must be directed toward the adverse effects of drug‐micronutrient interactions to protect patient health.

### 5.5 Future Directions in Micronutrient Research: Developing Systemic Precision Nutrition Strategies

Recent studies in nutrigenetics and nutrigenomics have demonstrated significant variability in individual responses to dietary macronutrients and micronutrients, primarily due to genetic diversity. Consequently, a one‐size‐fits‐all approach to dietary supplementation is not universally applicable [296]. The precision nutrition strategy offers a scientific foundation for personalised micronutrient supplementation by integrating multi‐dimensional data, including an individual's genetic background, microbiota composition, molecular biomarker profiles and lifestyle factors [297]. The rapid advancement of precision medicine has been driven by an in‐depth understanding of the pivotal role of genetic variation in disease pathogenesis and therapeutic responses. A growing body of evidence suggests that genetic variants can significantly affect how individuals respond to dietary macronutrients and micronutrients [298, 299]. Wilson et al. [300] demonstrated that hypertensive patients carrying the MTHFR rs1801133 TT genotype achieved a more significant reduction in blood pressure through riboflavin supplementation compared to standard antihypertensive drug therapy. Furthermore, numerous studies have established that genetic variants in vitamin D metabolism‐related genes are strongly linked to decreased levels of 25‐hydroxyvitamin D (25[OH]D), thereby elevating the risk of osteoporosis, sarcopenia, autoimmune disorders, malignancies and CVD [301]. Genetic variability plays a crucial role in shaping individual epigenetic responses to dietary bioactive compounds such as polyphenols, omega‐3 fatty acids and dietary fibre. For instance, polymorphisms in genes encoding key epigenetic regulators, including DNA methylation enzymes (e.g., DNMTs, MTHFR) and histone deacetylases (e.g., SIRTs), can alter the extent to which individuals respond to folate, polyphenols or omega‐3 fatty acids [302]. The cardioprotective benefits of these nutrients may differ across populations. Thus, effective precision nutrition requires an integrated approach that considers not only traditional micronutrients but also the complex interactions between dietary bioactive compounds and genetic variability.

Individual variability in response to nutritional interventions cannot be explained solely by genetic factors; multiple non‐genetic influences also play critical roles in shaping metabolic phenotypes and nutritional needs [260]. With ageing, both metabolism and nutritional requirements undergo profound changes [303]. Older adults typically experience reduced lean body mass and lower levels of physical activity, resulting in decreased basal energy expenditure [304]. Nevertheless, their demand for nutrients such as protein, vitamin D, vitamin B12 and calcium increases to support muscle maintenance, bone health and neurological function [305]. Besides, age‐related declines in gastrointestinal function often compromise nutrient absorption efficiency [306]. Geography and dietary habits also exert a significant influence on nutritional status. For instance, populations adhering to the Mediterranean diet (characterised by high consumption of olive oil, fish and nuts) generally achieve higher intakes of antioxidants and omega‐3 fatty acids, which contribute to lipid metabolism regulation and anti‐inflammatory epigenetic effects [307]. In contrast, the traditional East Asian diet, rich in soy and fermented foods, is associated with distinct gut microbiota structures and phytochemical metabolism patterns, influencing the bioavailability of compounds such as isoflavones [308]. In Africa, regional dietary patterns present particular challenges, with widespread risks of inadequate intake of calcium (affecting ~54% of the population), zinc (40%) and selenium (28%), especially in non‐North African and West African regions [309]. Diets high in phytic acid and low in animal protein further reduce the bioavailability of minerals such as iron [310]. These findings underscore the critical role of agricultural environments and dietary cultures in shaping nutrient availability. Furthermore, comorbid conditions such as CVD and type 2 diabetes further complicate nutritional needs [311]. Patients often require sodium‐restricted and low‐glycemic diets, while disease‐related inflammation and medication use (e.g., metformin impairing vitamin B12 absorption) may aggravate micronutrient deficiencies [312]. In addition, tissue repair and metabolic stabilisation phases increase the demand for zinc, magnesium and antioxidant nutrients [313]. Taken together, the development of nutritional strategies should holistically integrate age‐related physiological changes, region‐specific dietary exposures and the unique metabolic challenges posed by comorbidities to achieve truly personalised nutritional interventions.

When considering precision nutrition, it is essential not to overlook the complex interactions between nutrients from a systemic perspective. Although precision nutrition can significantly enhance individualization through genetic profiling, relying solely on a single‐gene–single‐nutrient paradigm still has inherent limitations. Therefore, integrating systems nutrition with precision nutrition strategies allows for a more comprehensive consideration of multidimensional factors, including an individual's genetic characteristics, metabolic status and lifestyle, enabling the establishment of personalised nutritional requirement models. In the future, multi‐omics sequencing combined with artificial intelligence can be employed to analyse the complex interaction networks among nutrients, genes and metabolic pathways, while further developing intelligent delivery systems to achieve precise regulation [314]. This systemic approach not only enables accurate customization of personalised intervention strategies but also dynamically tracks the cascade of physiological responses triggered by nutritional interventions and optimises intervention plans in real time, thereby providing a more scientific dietary solution for the prevention and treatment of chronic diseases.

## 6 Conclusions

The interrelationship between micronutrients and AS exhibits a highly complex network. Existing studies have elucidated in detail the specific mechanisms of action of a variety of single micronutrients (including vitamins A, C, D, E, K and minerals such as zinc and selenium) in developing AS, such as antioxidant, anti‐inflammatory and endothelial protection. Still, it is important to realise that these micronutrients never act in isolation in the organism but rather work together to influence the process of AS through a sophisticated network of interactions. The main limitation of current research is that most clinical trials still use single‐nutrient intervention strategies, ignoring the synergistic effects between nutrients. At the same time, existing nutritional recommendations often fail to adequately take into account individual differences, including factors such as genetic background, metabolic characteristics and disease state. Future research should focus more on systematic exploration. On the one hand, studies on the synergistic effects of multiple nutrients based on natural dietary patterns (e.g., DASH diet, Mediterranean diet) need to be carried out, and the preventive and therapeutic effects of micronutrient supplementation on AS need to be verified through high‐quality randomised controlled trials. On the other hand, genomics, metabolomics, microbiomics (e.g., nutrient metabolism function assessment analysis of intestinal flora), and other multi‐omics technologies should be integrated to gain a deeper understanding of nutrient interaction networks at the molecular level, laying the foundation for the development of more accurate nutritional assessment tools. Overall, there is still much room for exploration in the research on the association between micronutrients and AS. The shift in systematic research strategies will promote the leap from empirical nutritional recommendations to precision medicine for AS prevention and control, and provide a new paradigm of more scientific and effective nutritional interventions for the prevention and control of CVD.

## Author Contributions

**Yuxin Ouyang:** writing – original draft (equal). **Weiwei Jiang:** writing – original draft (equal). **Xiongquan Long:** writing – original draft (equal). **Peng Mao:** visualization (equal). **Pingping He:** funding acquisition (equal), writing – review and editing (equal). **Xinping Ouyang:** funding acquisition (equal), writing – review and editing (equal).

## Conflicts of Interest

The authors declare no conflicts of interest.

## Data Availability

Data sharing not applicable to this article as no datasets were generated or analysed during the current study.
